# Enhanced ADP-heptose-dependent NF-κB activation by *Helicobacter pylori* CagA through cortactin-Src-dependent tyrosine phosphorylation of IKKβ

**DOI:** 10.1093/femsml/uqaf049

**Published:** 2026-01-06

**Authors:** Irshad Sharafutdinov, Nicole Tegtmeyer, Barbara Friedrich, Michael Naumann, Steffen Backert

**Affiliations:** Department of Biology, Division of Microbiology, Friedrich-Alexander-Universität Erlangen-Nürnberg, 91058 Erlangen, Germany; Department of Biology, Division of Microbiology, Friedrich-Alexander-Universität Erlangen-Nürnberg, 91058 Erlangen, Germany; Department of Biology, Division of Microbiology, Friedrich-Alexander-Universität Erlangen-Nürnberg, 91058 Erlangen, Germany; Institute of Experimental Internal Medicine, Medical Faculty, Otto-von-Guericke University Magdeburg, 39120 Magdeburg, Germany; Department of Biology, Division of Microbiology, Friedrich-Alexander-Universität Erlangen-Nürnberg, 91058 Erlangen, Germany

**Keywords:** ADP-heptose, CagA, cortactin, FAK, *Helicobacter pylori*, interleukin-8, inflammation, NF-κB, Src, T4SS, TIFA

## Abstract

Nuclear factor kappa-light-chain-enhancer of activated B cells (NF-κB) represents a family of important transcription factors in innate immunity. We have previously reported that the gastric pathogen *Helicobacter pylori* needs the actin-binding protein cortactin for efficient interleukin-8 (IL-8) secretion, which requires NF-κB activation. However, it remained unknown, which exact cortactin signaling mechanism contributes to IL-8 release. In fact, *H. pylori* profoundly activates NF-κB in wild-type AGS gastric epithelial cells by the effector molecule adenosine diphosphate (ADP)-β-d-manno-heptose (ADPH) in a type IV secretion system-dependent manner. However, the injected CagA protein might contribute to NF-κB activation. The ADPH-stimulated canonical NF-κB cascade involves alpha-kinase 1 and adapter protein TRAF-interacting protein with forkhead-associated domain (TIFA) to activate inhibitor of kappa B (IκB) kinases (IKKs), followed by phosphorylation-dependent degradation of IκBα and subsequent nuclear translocation of p65 NF-κB and IL-8 release. Here, we show that infection of cortactin knockout cells leads to reduced activation of focal adhesion kinase (FAK) and c-Sarcoma (Src) kinase resulting in diminished phosphorylation of IKKβ at tyrosine residue 199 and subsequently phosphorylation of p65 at serine residue 536, both of which are associated with downregulated NF-κB activity. Our results were further supported using FAK and TIFA knockout cells and treatments with purified ADPH and overexpression of CagA, showing cumulative effects in wild-type, but not in knockout cells. These data demonstrate that ADPH-dependent NF-κB activation and IL-8 secretion are enhanced by CagA. Together, we present here a novel CagA>cortactin>FAK>Src>IKKβ signaling cascade, contributing to proinflammatory responses by *H. pylori*.

## Teaser

Identification of a novel type-IV secretion-dependent CagA>cortactin>FAK>Src>IKKβ signaling cascade in gastric epithelial cells, contributing to canonical adenosine diphosphate (ADP)-heptose-mediated nuclear factor kappa-light-chain-enhancer of activated B cells (NF-κB) activation by *Helicobacter pylori*. This study investigates how two *H. pylori* effector molecules, ADP-heptose and CagA, can regulate and modulate proinflammatory transcription factor NF-κB. The data demonstrate that the major ADP-heptose-dependent NF-κB activity is enhanced by CagA through activation of a novel CagA>cortactin>FAK>Src>IKKβ signaling cascade that leads to pronounced phosphorylation and nuclear translocation of p65 NF-κB and elevated interleukin-8 secretion.

## Introduction

*Helicobacter pylori* is a major gastric pathogen that chronically infects the human stomach and plays a central role in the development of severe gastrointestinal disorders, ranging from gastritis and peptic ulcer disease to gastric cancer and mucosa-associated lymphoid tissue lymphoma (Hatakeyama 2019). *Helicobacter pylori* employs a complex virulence machinery that hijacks host cell signaling pathways, leading to deregulated cellular responses and the development of gastric pathology. Urease is secreted by *H. pylori* to convert urea into ammonia and carbon dioxide, which helps the bacterium to neutralize gastric acid and survive in the highly acidic environment of the stomach (Stingl et al. 2002, Graham and Miftahussurur 2018). Outer membrane proteins such as the blood group antigen-binding adhesin (BabA), the sialic acid-binding adhesin (SabA), the *Helicobacter* outer protein Q (HopQ), and the outer inflammatory protein A (OipA) facilitate adhesion of the bacterium to epithelial and other cell types, and contribute to disturbance of host cell signaling (Loh et al. 2008, Su et al. 2016). In addition, serine protease HtrA and major intracellular effectors such as ADPH, cytotoxin-associated gene A (CagA) and vacuolating cytotoxin A (VacA) compromise epithelial cell integrity and target host signaling pathways (Backert and Tegtmeyer 2017, Chauhan et al. 2019, Pfannkuch et al. 2019). In particular, translocated CagA can be phosphorylated by Src and Abl tyrosine kinases at its tyrosine residues 899 (Y-899), 918 (Y-918), and/or 972 (Y-972) involved in cytoskeletal remodeling of host cells (Mueller et al. 2012). In addition, *H. pylori* infection triggers nuclear factor kappa-light-chain-enhancer of activated B cells (NF-κB) signaling that plays a crucial role in the host’s immune response and inflammation (Naumann et al. 2023). In fact, *H. pylori*-mediated activation of the NF-κB transcription factor complex triggers the expression and release of proinflammatory cytokines, such as interleukin-8 (IL-8) (Gong et al. 2010, Sokolova et al. 2013). In the context of *H. pylori* infection, IL-8 overexpression is generally associated with pronounced inflammatory processes and consequently a higher risk of gastric cancer development (Sokolova and Naumann 2017).

Two bacterial factors, ADPH and CagA, were both shown to regulate NF-κB activity (Naumann et al. 2023). ADPH, a derivative of d-glycero-β-d-manno-heptose 1,7-bisphosphate (βHBP) in the lipopolysaccharide biosynthesis cascade, is a molecule produced by Gram-negative bacteria, which was identified as a novel pathogen-associated molecular pattern (PAMP) (Gall et al. 2017, Stein et al. 2017, Zimmermann et al. 2017, Pfannkuch et al. 2019). In fact, isolated ADPH is capable of activating both canonical and noncanonical NF-κB pathways via alpha-kinase 1 (ALPK1) signaling to either TIFA/TRAF6/TGFβ‐activated kinase 1 (TAK1) or TIFA/TRAF2/NF‐κB‐inducing kinase (NIK), respectively (Maubach et al. 2021). Mechanistically, ALPK1/ADPH-dependent TIFA phosphorylation at threonine 9 (T-9) is required for the downstream oligomerization of TRAF6 and the activation of the canonical NF-κB pathway (Milivojevic et al. 2017, Zhou et al. 2018). The complexes formed by TIFA (TRAF6 in canonical NF-κB and TRAF2 in noncanonical NF-κB), also known as TIFAsomes, were shown both activated by *H. pylori* ADPH (Maubach et al. 2021). A recent study showed that upon *H. pylori* infection, the deubiquitinylase cylindromatosis (CYLD) promotes canonical NF-κB pathway via constitutive interaction with TRAF6, which prevents the deubiquitinylation of the latter by the deubiquitinylase A20 (Lim et al. 2025). Of the two kinases, NIK-activated inhibitor of kappa B (IκB) kinase (IKKα) mediates formation of p52/RelB in the noncanonical manner, while TAK1-phosphorylated IKKβ is responsible for the activation of p50/p65 complex in the canonical NF‐κB pathway (Chen and Greene 2004). IKKβ plays a key role in activation of NF-κB via phosphorylation of IκBα, leading to its ubiquitinylation and proteasomal degradation (Stephenson et al. 2023). IκBα degradation releases the NF-κB complex of p50/p65, allowing its translocation into the nucleus and activation of target genes involved in inflammation, immune responses, and cell survival. IKKβ is regulated through serine phosphorylation at S-177 and S-181, and tyrosine phosphorylation at residues Y-188 and Y-199 (Huang et al. 2003). *Helicobacter pylori* infection was shown to induce IKKβ tyrosine phosphorylation at Y-199 via transient binding of c-Sarcoma kinase (Src), which enhanced the phosphorylation of IκBα as well as p65 (Rieke et al. 2011). The p65 phosphorylation at serine residue 536 (S-536) was shown to enhance the transactivation potential of NF‐κB (Stephenson et al. 2023). Furthermore, early studies from various groups have reported that NF-κB activation and IL-8 release by *H. pylori* also somehow involve CagA, at least partially (Crabtree et al. 1995, Sharma et al. 1995, Yamaoka et al. 1996, Ando et al. 2000, Papadakos et al. 2013, Zhang et al. 2015). Additionally, it was shown that transfection of CagA induces the production of IL-8 in human gastric adenocarcinoma (AGS) cells (Brandt et al. 2005, Kim et al. 2006), which required activation of the CagA>Ras>Raf>MEK>ERK1/2>NF-κB signaling cascade (Brandt et al. 2005). In agreement with these observations, IL-8 induction by *H. pylori* was sensitive to pharmacological inhibition of the ERK1/2 kinases (Keates et al. 1999, Nozawa et al. 2002). However, the exact link between CagA-mediated ERK1/2 stimulation and NF-κB activation remained unknown.

Recently, we discovered that the host cell protein cortactin can in some way support inflammatory NF-κB signaling by various bacterial pathogens (Tegtmeyer et al. 2022). Cortactin is an actin-binding protein that regulates cytoskeletal dynamics through its interactions with the Arp2/3 complex, F-actin, and various signaling proteins via its N-terminal acidic, proline-rich region and a C-terminal Src homology 3 (SH3) domains (Schnoor et al. 2018). Given its key role in cytoskeletal dynamics, it is not surprising that cortactin is targeted by many microbial pathogens including *H. pylori* (Sharafutdinov et al. 2022, 2024a, Selbach and Backert 2005). Furthermore, another actin cytoskeleton remodeling protein, WAVE3, was previously shown to modulate the NF-κB activity in cancer cells, and WAVE3 colocalized with cortactin in invadopodia (Davuluri et al. 2014). We have previously shown that cortactin is essential for activation of focal adhesion kinase (FAK), Src and Abl as well as efficient CagA phosphorylation (Knorr et al. 2021). Given the fact that Src kinase can also phosphorylate IKKβ at Y-188 and Y-199 (Huang et al. 2003), we hypothesized that cortactin might mediate IKKβ kinase phosphorylation via FAK/Src pathway during *H. pylori* infection. Therefore, in this work we aimed to elucidate (1) whether cortactin plays a role in IKKβ phosphorylation upon infection and (2) whether injected CagA contributes to ADPH-induced NF-κB activation.

## Materials and methods

### Host cell lines

For infection and transfection experiments, the human adenocarcinoma cell line AGS (#CRL-1739, ATCC, Manassas, VA, USA) wild-type (wt) and various knockout variants were used. These AGS cells were grown at 37°C and 5% CO_2_ in RPMI 1640 medium (Gibco, Darmstadt, Germany) supplemented with 10% fetal calf serum (Gibco, Paisley, UK), 1% antibiotic mix penicillin/streptomycin (Sigma-Aldrich, Steinheim, Germany), and 0.2% Normocin® (InvivoGen, Toulouse, France). The AGS cell line with the cortactin gene knockout was generated previously using the CRISPR/Cas9 plasmids (#sc-400761, Santa Cruz Biotechnology, Heidelberg, Germany) and the HDR-plasmid (#sc-400761-HDR, Santa Cruz Biotechnology) as described (Knorr et al. 2021). The AGS cell line with *fak* gene knockout was generated using the CRISPR/Cas9 plasmids (#sc-400089-KO-2, Santa Cruz Biotechnology) and the HDR-plasmid (#sc-400089-HDR2, Santa Cruz Biotechnology). The AGS cell knockout for *tifa* was generated previously (Gall et al. 2017), and kindly provided by Dr Nina Salama (Fred Hutchinson Centre, Seattle, USA). Primary wt and cortactin knockout mouse embryonic fibroblast (MEF) cells were published previously (Lai et al. 2009) and kindly provided by Dr Klemens Rottner (Helmholtz Centre for Infection Research, Braunschweig, Germany).

### Bacterial strains and infection experiments

The *H. pylori* strains used for the infection experiments included *H. pylori* P12 wt, and the P12Δ*cag*PAI, P12Δ*cagY*, P12Δ*cagA*, and P12Δ*gmhA* deletion mutants (Pachathundikandi et al. 2019, Tegtmeyer et al. 2020, Maubach et al. 2021). The bacteria were routinely cultured from the glycerol stocks onto GC agar plates supplemented with 10% horse serum (PAN-Biotech GmbH, Aidenbach, Germany), 10 µg/ml vancomycin (Sigma-Aldrich, St. Louis, MO, USA), and 4 µg/ml amphotericin (Sigma-Aldrich). The GC agar plates used for the growth of the isogenic *H. pylori* mutant strains additionally contained appropriate antibiotics. The bacteria were allowed to grow for 48 h in microaerophilic conditions provided by 2.5 l anaerobic jar (Oxoid, Wesel, Germany) with a CampyGen gas mix pouches (Oxoid) (Moese et al. 2001). For infection experiments, bacterial suspensions were prepared in BHI medium (Oxoid). 16 h prior infection, AGS cells were washed three times with phosphate buffered saline (PBS) buffer and were further incubated in antibiotic-free medium. The infections were performed at a multiplicity of infection of 50 at the indicated time points. After infection, AGS cells were harvested for either Western blotting or immunofluorescence microscopy.

### Transfection of AGS and MEF cells and treatment with purified ADPH

AGS cells and MEFs were transfected with plasmid DNA constructs expressing CagA (Brandt et al. 2005), GFP-cortactin wt and S405/418A mutant (Tegtmeyer et al. 2011), NF-κB p65 (Clontech) or IL-8 (Dragneva et al. 2001), tagged with yellow fluorescent protein (YFP) and green fluorescent proteins (GFP), respectively, using the Turbofect transfection reagent (Thermo Fisher Scientific, Waltham, MA, USA). While NF-κB p65-YFP is expressed from the constitutive CMV promoter, the IL-8 gene in the pIL-8-GFP plasmid is controlled by the inducible IL-8 promoter sequences +420 to +102 (Dragneva et al. 2001). After 24-h transfection, the cells were washed with plain Roswell Park Memorial Institute (RPMI) medium followed by *H. pylori* infection or treatment with adenosine diphosphate (ADP)-heptose (#tlrl-adph, InvivoGen) as described (Maubach et al. 2021).

### SDS-PAGE and immunoblot analysis

To analyse protein expression levels, samples were harvested by using hot SDS buffer, boiled for 5 min, and then subjected to standard dodecyl sulfate polyacrylamide gel electrophoresis (SDS-PAGE). After SDS-PAGE, the samples were blotted on polyvinylidene difluoride Immobilon-P membranes (Merck-Millipore, Darmstadt, Germany). Next, the membranes were blocked for 1 h with TBS-T buffer (25 mM Tris–HCl pH 7.4, 140 mM NaCl, 0.1% Tween-20) containing either 5% skim milk or 3% bovine serum albumin. For immunodetection of nonphosphorylated and phosphorylated CagA, primary polyclonal α-CagA antibody (#HPP-5003–9, Austral Biologicals, San Ramon, CA, USA) and monoclonal α-pan-phosphotyrosine antibody PY-99 (#sc-7020, Santa Cruz Biotechnology) were used respectively. Other antibodies used in this work were specific for detecting CagY (Tegtmeyer et al. 2020), UreB (Tegtmeyer et al. 2013), cortactin (#05–180, Merck-Millipore), Src (#2123, Cell Signaling Technology, Frankfurt, Germany), GAPDH (#sc-47724, Santa Cruz Biotechnology), TIFA (#61358, Cell Signaling Technology), phospho-TIFA-T9 (#ab214815, Abcam, Cambridge, UK), IKKβ (#05–535, clone 10AG2, Merck-Millipore), phospho-IKKβ-Y199 (#59195, Abcam), phospho-NF-κB p65-S536 (#3031, Cell Signaling Technology), FAK (#610087, BD Bioscience, Heidelberg, Germany), phospho-FAK-Y397 (#3283S, Cell Signaling Technology), and phospho-Src-Y418 (#OP07, Oncogene, Heidelberg, Germany). α-rabbit (#31462) or α-mouse (#31446) polyvalent goat antibodies conjugated with horseradish peroxidase (HRP) were used as secondary antibodies (Thermo Fisher Scientific). Due to low cellular expression of TIFA and TIFA phospho-proteins (T-9), we used immunoprecipitation (IP) assay followed by Western blotting analysis as described earlier (Tegtmeyer et al. 2011). The detection of antibodies was performed by using the ECL Prime chemiluminescence Western blot kit (GE Healthcare) as described previously (Blumenthal et al. 2007).

### Quantification of Western blot protein bands

To quantify the protein expression levels obtained by Western blotting, the Image Lab software (BioRad, Munich, Germany) was used for the densitometric analysis of the immunoblot band intensities. The protein expressions were normalized to the GAPDH expression blot and shown as fold change of expression relative mock control, which was set as 1.

### Fluorescence microscopy

For the fluorescence microscopy, AGS wt and AGSΔ*cttn* cells were grown on glass coverslips in 12-well plates in RPMI medium, as described above. 24 h prior infection, fetal calf serum (FCS) was removed from the AGS culture medium in order to prevent NF-κB activation by growth factors. After 8-h infection, cells were fixed in 4% paraformaldehyde for 10 min. Before staining, cells were permeabilized with 0.25% Triton-X-100 for 10 min. For immunostaining of cortactin, mouse α-cortactin (#05–180, Merck-Millipore) was used as a primary antibody. For analysis of activated NF-κB p65, cells were stained with rabbit α-phospho-NF-κB p65 (#3031, Cell Signaling Technology), detecting NF-κB p65 only when phosphorylated at serine 536. Fluorescein isothiocyanate-conjugated goat α-rabbit antibodies were used as secondaries. Optionally, cells were counterstained with rhodamine-phalloidin (#R415, Thermo Fisher Scientific, Darmstadt, Germany) and 4’-6-diamidino-2-phenylindole dihydrochloride (DAPI, from Thermo Fisher Scientific) to visualize filamentous actin and cell nuclei, respectively. Samples were analysed under the Leica Stellaris 8 fluorescence microscope (Leica Microsystems, Wetzlar, Germany). Imaging was performed in sequential mode with excitation/emission values of 405/430–479 for DAPI, 488/496–514 for GFP, 514/521–535 for YFP, and 551/595–636 for rhodamine via LAS AF computer software (Leica Microsystems).

### Quantification of fluorescence intensity

To assess fluorescence intensities of phospho-NF-κB p65-S536, NF-κB p65-YFP, and IL-8-GFP, the images were analysed with the ImageJ software (Schindelin et al. 2012). Fluorescence levels of nuclear NF-κB p65 were assessed by segmentation of DAPI-stained nuclei into the regions of interest (ROIs) as previously described (Sharafutdinov et al. 2020a). The selected ROIs were further used for quantification of fluorescence intensities of NF-κB p65. Similarly, ROIs were defined for quantification of IL-8 fluorescence intensities. Subsequently, fluorescence intensities were expressed as relative fluorescence units (RFU), obtained by dividing the total fluorescent signal of ROI by area of ROI.

### Secreted embryonic alkaline phosphatase reporter assay

In order to analyse relative NF-κB activity, we performed the secreted embryonic alkaline phosphatase (SEAP) reporter assay as described previously (Tegtmeyer et al. 2020). AGS cells grown in 6-well plates were transfected with the pNF-κB-SEAP reporter plasmid (http://www.addgene.org) as described above. To assess the CagA potential on NF-κB activation, AGS cells were additionally transfected with the CagA-expressing construct. Afterwards, the cells were either infected with the indicated *H. pylori* strains or treated with purified ADPH. To quantify the NF-κB-dependent SEAP production, 20 μl of cell culture medium were mixed with 180 μl of QUANTI-Blue™ reagent (InvivoGen) in 96-well plates. After incubation for 30 min at 37°C, the measurement of the optical density at 620 nm was performed at the Infinite F200 Pro microplate reader (Tecan, Männedorf, Switzerland).

### IL-8 and KC ELISA

In order to study the production and release of IL-8 (corresponding to KC in mice), supernatants of infected or treated cells were subjected to ELISA by using commercial kits according to the manufacturer’s instructions (#DY453-05 from R&D Systems, Minneapolis, MN, USA and #88–8086 from Thermo Fisher Scientific). In brief, ELISA plates were coated overnight at 4°C with 100 μl/well capture antibody. After washing the plates three times with 250 μl/well wash buffer, the wells were blocked with assay diluent and incubated at room temperature (RT) for 1 h. Following another washing step, the supernatants were diluted 1:10 in assay diluent and 100 μl/well of the samples were added to the corresponding wells and incubated for 2 h at RT. After washing the plates three times, 100 μl/well of the detection antibody was added and incubated for 1 h at RT. Subsequently, 100 μl/well of avidin HRP was added after the plates had been washed three times again. After 30 min incubation at RT, three more washing steps followed and 100 μl/well of the substrate solution tetramethylbenzidine were added for 15 min at RT. Finally, 100 μl/well of stop solution (2 N H_2_SO_4_) were added and plates were measured at 450 nm using a TECAN Infinite 200 PRO plate reader.

### Statistics

Experiments were performed independently at least three times. Statistics was performed by using Student’s *t*-test. Statistical significance was considered significant with *P*-values *P* ≤ .05 (*), *P* ≤ .01 (**), and *P* ≤ .001 (***) or nonsignificant (n.s.) with *P*-values *P* > .05.

## Results

### Efficient NF-κB activation by *H. pylori* requires cortactin, ADPH, and CagA

Cortactin was recently shown to play a role in IL-8 secretion by *H. pylori, Salmonella enterica*, and *Pseudomonas aeruginosa*, but not *Campylobacter* spp. (Tegtmeyer et al. 2022). In particular, cortactin-dependent IL-8 secretion required efficient activation of the Src kinase (Tegtmeyer et al. 2022). Therefore, we were interested to study the role of cortactin in the NF-κB activation in more detail. Here, we used the parental gastric epithelial cell line AGS wt and the cortactin deletion mutant (AGSΔ*cttn*) generated previously by CRISPR-Cas9 (Knorr et al. 2021). Cortactin expression was analysed by Western blot (Fig. S1A) and immunofluorescence microscopy (Fig. S1B) and was expectedly observed in AGS wt, but not in the AGSΔ*cttn* knockout cells. As another control, the expression of Src kinase was similar in both parental and AGSΔ*cttn* cells (Fig. S1A). In addition, we validated the expression of selected bacterial proteins in wt *H. pylori* and isogenic mutant strains as control (Fig. S1C). The Δc*ag*PAI and Δ*cagY* deletion mutant strains expectedly showed no expression of either CagY and/or CagA proteins, confirming defective type IV secretion system (T4SS) functions. The *H. pylori* ADPH mutant strain (Δ*gmhA*) could produce T4SS-associated CagA and CagY proteins (Fig. S1C), but was defective for ADPH-dependent responses upon infection (Maubach et al. 2021).

**Figure 1.**
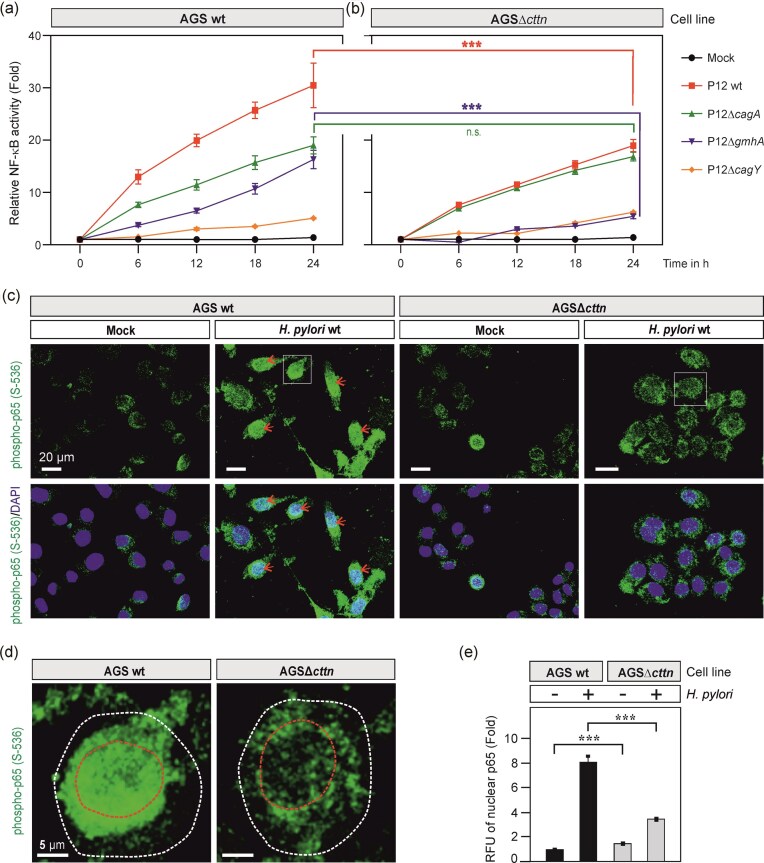
Cortactin gene knockout results in reduced activation of NF-κB associated with reduced phospho-NF-κB p65 (S-536) recruitment into AGS cell nuclei upon *H. pylori* infection. Time course of NF-κB activation over 24 h in (A) AGS wt and (B) AGSΔ*cttn* knockout cells during infection with the indicated *H. pylori* strains. Quantification of NF-κB activity by SEAP reporter assay (Quanti Blue) reveals enhanced NF-κB activity in wt vs. Δ*cttn* AGS cells, which depends on the functional *cag* type IV secretion system and the effector molecules ADPH and CagA. (C) AGS cells were incubated without (Mock) or with *H. pylori* for 4 h, and then stained for phospho-NF-κB p65 (S-536) (green) and nuclei (blue). As revealed by fluorescence microscopy, *H. pylori* provoke strong activation of NF-κB p65 in wt (red arrowheads), but not in Δ*cttn* cells. (D) Representative AGS wt and Δ*cttn* cells from panel A (cells in white boxes) showing phospho-NF-κB p65 accumulation (green) within the nuclei upon infection with wt *H. pylori*. Nuclear and cellular margins are indicated by red and white dashed lines, respectively. (E) Relative fluorescence intensity (RFU) of nuclear phospho-NF-κB p65 was quantified. Mean values ± standard error are shown with significant difference *** corresponding to *P* ≤ .001.

In order to investigate whether cortactin is involved in NF-κB activation, we transfected AGS wt and AGSΔ*cttn* cells with a SEAP reporter plasmid to monitor NF-κB activity, followed by infection with the above *H. pylori* strains in a time course of 6, 12, 18, and 24 h (Fig. 1A and B). The results show that infection of AGS wt cells with wt *H. pylori* induced profound and continuously growing NF-κB activity until the 24 h time point (30.5-fold), which was significantly lower in the infected AGSΔ*cttn* cells (19-fold) under the same conditions. However, infection with the T4SS-deficient Δ*cagY* mutant showed nearly basal levels of NF-κB activity both in AGS wt and AGSΔ*cttn* cells, suggesting that its effector(s) might play a role (Fig. 1A and B). Similar data were observed for the mutant lacking the entire *cag*PAI (data not shown). Interestingly, the ADPH mutant Δ*gmhA* revealed a statistically significant 16-fold vs. 5-fold induction of NF-κB in AGS wt and AGSΔ*cttn* cells, respectively, while the Δ*cagA* mutant exhibited similar moderate NF-κB activation rates (19-fold vs. 17-fold) in both cell lines, which were statistically not significant (Fig. 1A and B). These data suggest that the host factor cortactin and the bacterial T4SS-effectors ADPH and CagA are involved in *H. pylori*-triggered NF-κB activation.

### Pronounced nuclear translocation of p65/NF-κB and IL-8 production by *H. pylori* requires cortactin

Previous studies demonstrated that NF-κB activation in response to *H. pylori* infection requires phosphorylation of the NF-κB p65 subunit, followed by its translocation into the cell nucleus (Keates et al. 1997). In order to investigate further how cortactin is involved in NF-κB activation, AGS wt and AGSΔ*cttn* cells infected with wt *H. pylori* for 4 h were stained with a phospho-specific p65 antibody (recognizing p65 protein only when phosphorylated at S-536), and counterstained with DAPI to visualize the cell nuclei. Infection with *H. pylori* increased nuclear levels of phospho-p65 in AGS wt cells (Fig. 1C, red arrows), while the fluorescence intensity of nuclear phospho-p65 in AGS∆*cttn* was noticeably less (Fig. 1C, right panels). Almost all AGS wt cells demonstrated increased fluorescence of nuclear phospho-p65 in comparison to AGS∆*cttn*, which can be seen in enlarged representative images (Fig. 1D). We then quantified the RFUs of nuclear phospho-p65 in AGS cells. Interestingly, noninfected AGSΔ*cttn* cells demonstrated slightly higher p65 fluorescence intensity, compared to AGS wt, though the effect was not pronounced (Fig. 1E). In turn, infection with *H. pylori* resulted in an eight-fold increase of the nuclear phospho-p65 in AGS wt cells. Interestingly, infection of AGS∆*cttn* cells also resulted in approximately four-fold increase of p65 fluorescence intensity, indicating that another, cortactin-independent activation of p65 also exists.

**Figure 2.**
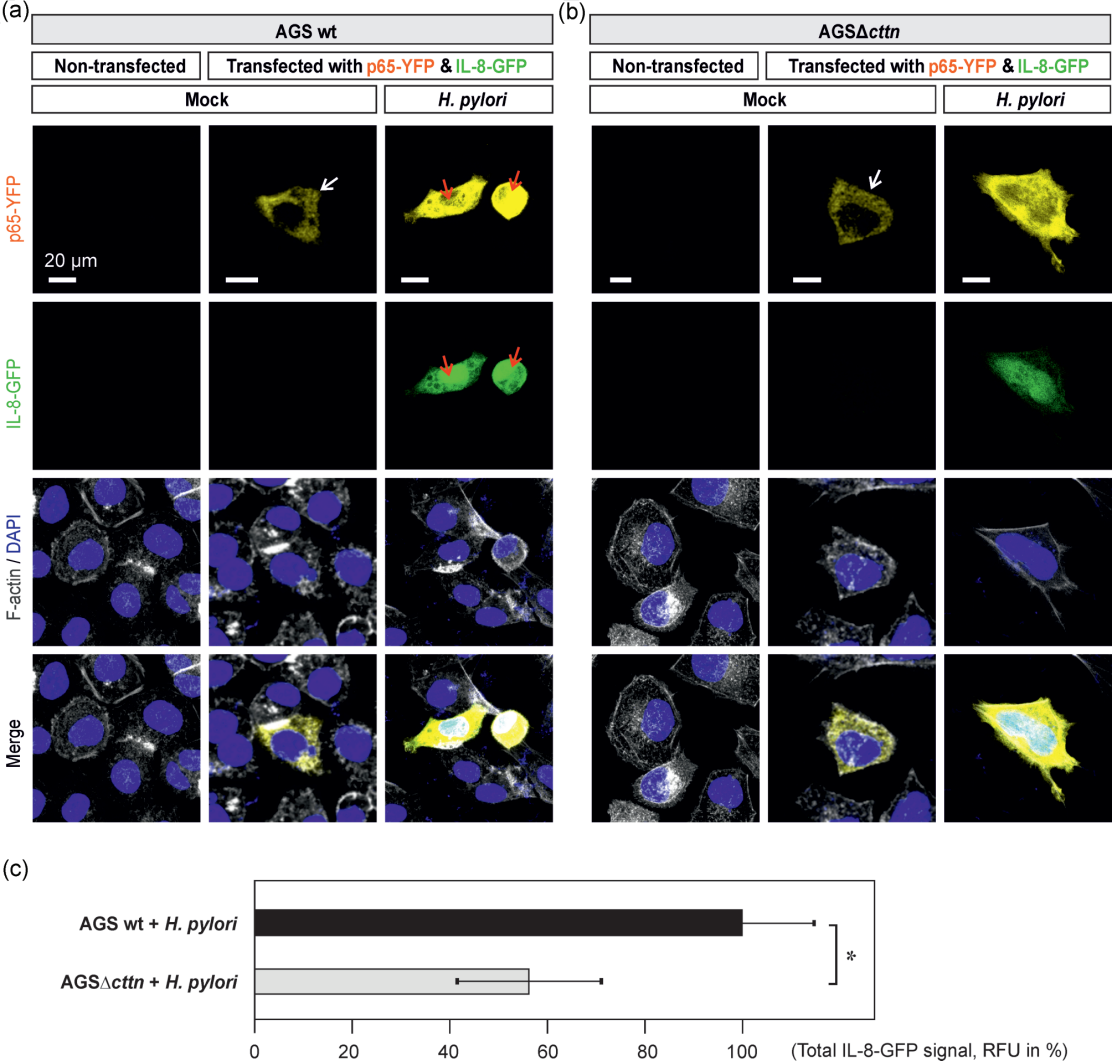
AGS cells with cortactin deficiency demonstrate diminished NF-κB p65 and IL-8 cellular levels in response to *H. pylori* infection. (A) AGS wt and (B) AGSΔ*cttn* knockout cells were transfected with pNF-κB p65-YFP (yellow) and pIL-8-GFP (green) constructs for 24 h followed by 4 h incubation without (mock) or with wt *H. pylori*. After fixation, cells were stained with DAPI and phalloidin to visualize cell nuclei (blue) and filamentous actin (gray), respectively. White arrows indicate cellular expression of NF-κB p65-YFP in the absence of *H. pylori*, while red arrows show increased levels of NF-κB p65-YFP and pIL-8-GFP in the presence of the pathogen. (C) RFU of pIL-8-GFP in transfected AGS wt vs. Δ*cttn* cells were quantified. The values in AGS wt cells were set to 100%. Mean values ± standard error are shown with significant difference * corresponding to *P* ≤ .05.

To study the role of cortactin in NF-κB activation in more detail, we next performed transfection of the AGS cell variants with constructs expressing p65-YFP and inducible IL-8-GFP. Transfected AGS wt and AGSΔ*cttn* cells were infected with *H. pylori* for 4 h, followed by counterstaining of cell nuclei and filamentous actin. Fluorescence microscopy revealed that both AGS wt and AGSΔ*cttn* cells in the absence of *H. pylori* can express similar levels of p65-YFP (Fig. 2A and B, white arrows). However, when exposed to the bacteria, AGS wt cells demonstrated considerably higher fluorescence intensities both of p65-YFP and IL-8-GFP (Fig. 2A, red arrows), compared to AGSΔ*cttn* cells (Fig. 2B). Expectedly, RFU quantification revealed that *H. pylori* infection resulted in IL-8-GFP expression in AGS wt cells. On the other hand, infected AGSΔ*cttn* cells showed diminished IL-8-GFP expression with about 43% decrease compared to AGS wt cells (Fig. 2C). Therefore, both immunostaining with phosphospecific antibodies and transfection experiments indicate a functional role of cortactin in NF-κB activation upon *H. pylori* infection.

### Cortactin enhances T4SS-dependent IKKβ tyrosine phosphorylation during *H. pylori*-induced NF-κB and IL-8 activation

**Figure 3.**
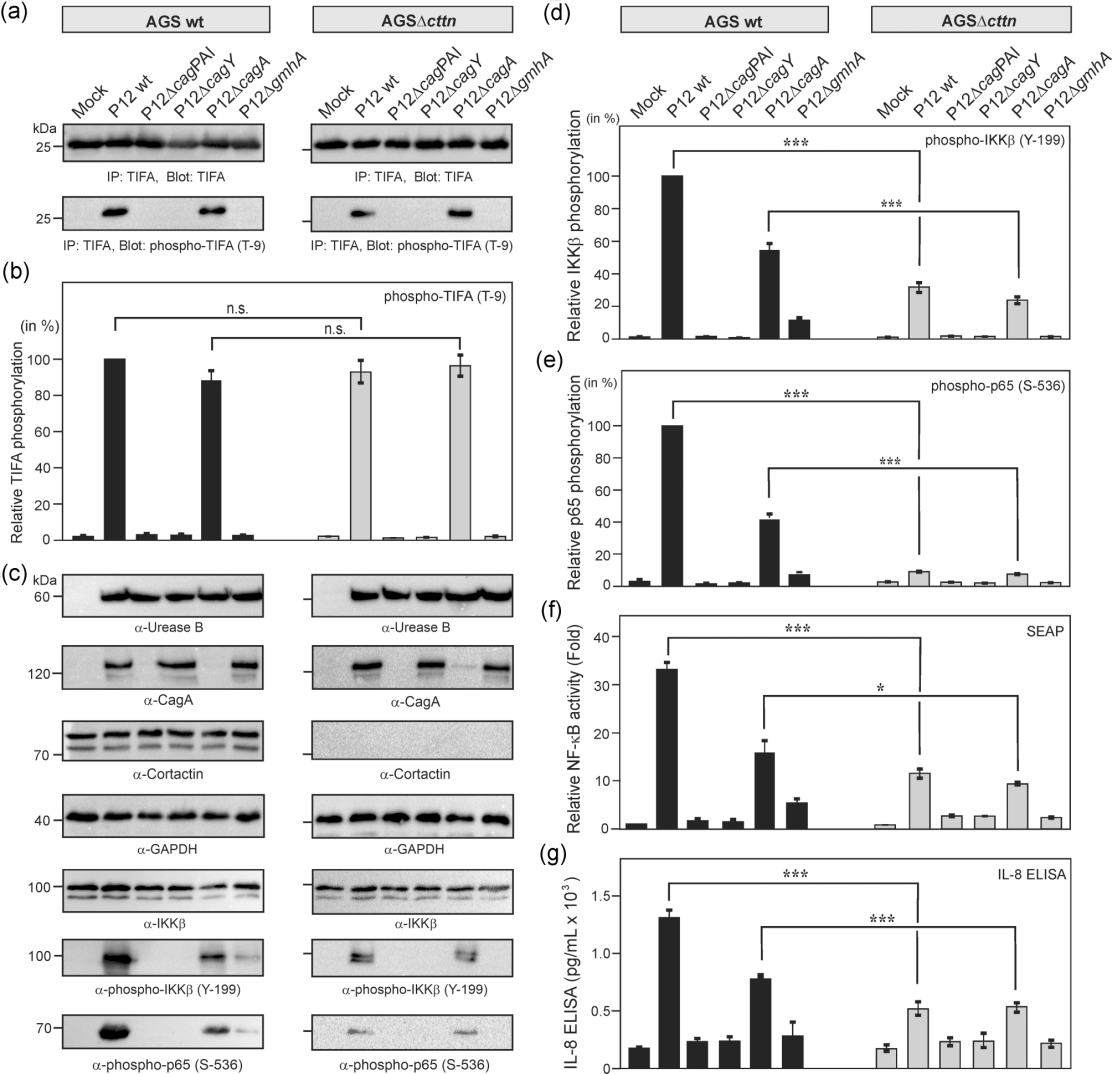
TIFA and NF-κB signaling activation in AGS cells upon *H. pylori* infection. (A) AGS wt or AGSΔ*cttn* knockout cells were infected with the indicated *H. pylori* strains for 45 min. Protein lysates were subjected to IP with anti-TIFA antibodies. IP samples against total TIFA show equal amounts of TIFA present across all samples. Probing for phospho-TIFA (Threonine 9) demonstrates activation of TIFA in *H. pylori* wt and Δ*cagA* mutant-infected samples (lanes 2 and 5), while no activation is observed in mock, Δ*cag*PAI, Δ*cagY*, or Δg*mhA* infections (lanes 1, 3, 4, and 6). (B) Quantification of TIFA phospho-band intensities from panel A. (C) Total protein lysates prepared from infected cells after 45 min were subjected to Western blotting against bacterial proteins (Urease B and CagA) as well as host cell total cortactin, IKKβ and GAPDH, which confirms similar expression levels across all samples. Phospho-specific blots against IKKβ (Y-199) and p65 (S-536) revealed their pronounced activation only in lanes 2 and 5, as expected. Quantification of phospho-IKKβ (D) and phospho-p65 (E) band intensities of panel C. The data are presented as relative band intensity values. (F) Quantification of NF-κB activity by SEAP reporter assay (Quanti Blue) and (G) IL-8 secretion by standard ELISA in the same set of experiments. These studies revealed increased NF-κB activity and IL-8 production in cells infected for 6 h with *H. pylori* wt and Δ*cagA* mutant strains. Mean values ± standard error are shown with significant difference *** corresponding to *P* ≤  .001 and * to ≤ .05.

Next, we aimed to identify how cortactin knockout cells affect T4SS-, *gmhA*-, or *cagA*-dependent NF-κB signaling upon infection. It was recently shown that ADPH-dependent TIFA phosphorylation and downstream signaling to NF-κB activation, such as phosphorylation of IKKβ and p65, occurs early upon *H. pylori* infection within the first 60 min (Rieke et al. 2011, Maubach et al. 2021). In addition, ADPH-dependent TIFA degradation is starting after 1 h (Maubach et al. 2021, Snelling et al. 2022). Thus, we infected AGS wt and AGSΔ*cttn* cells with the designated strains for 45 min and the protein lysates were subjected to IP and/or Western blotting, and then probed with the indicated antibodies. ADPH-dependent TIFA phosphorylation at threonine residue 9 (T-9) is a hallmark of the canonical NF-κB pathway activation, which leads to TIFAsome formation (Zimmermann et al. 2017, Zhou et al. 2018, Maubach et al. 2021). IPs of TIFA after 45 min showed equal amounts of TIFA present in all samples (Fig. 3A). Further probing of these IPs against phospho-TIFA showed TIFA phosphorylation at T-9 by *H. pylori* wt and Δ*cagA* mutant in both AGS wt and AGSΔ*cttn* cells. In contrast, no TIFA activation was observed in either Δ*cag*PAI, Δ*cagY* or Δ*gmhA* infections, indicating a major role of T4SS- and ADPH-dependent ALPK1/TIFA regulation in mediating NF-κB activation. Quantification of phospho-TIFA (T-9) bands showed similar levels of TIFA activation both in AGS wt and AGSΔ*cttn* cells infected with either *H. pylori* wt or Δ*cagA* mutant, suggesting that neither cortactin nor CagA are involved in the initial steps of NF-κB activation upstream of TAK1 (Fig. 3B).

Control Western blots of AGS cell lysates with attached bacteria expectedly showed equal amounts of urease B in all strains and similar CagA expression where expected (Fig. 3C), indicating similar binding capabilities of the bacteria. Comparable CagA expression was observed by *H. pylori* wt as well as the Δ*cagY* and Δ*gmhA* deletion mutants, with no expression in the Δ*cag*PAI or Δ*cagA* deletion strains as expected (Fig. 3C, top). After 45 min of infection, phospho-IKKβ (Y-199) and phospho-p65 (S-536) were induced downstream of phospho-TIFA (T-9) in AGS wt cells infected by *H. pylori* wt and to a lesser extent in the Δ*cagA* mutant (Fig. 3C, lanes 2 and 5), indicating a role of CagA in this process. Quantification of the band intensities on Western blots confirmed that phosphorylation of IKKβ (Y-199) and p65 (S-536) is enhanced by CagA in a cortactin-dependent fashion (Fig. 3D and E). Furthermore, in AGSΔ*cttn* infected cells, phosphorylation of IKKβ (Y-199) and p65 (S-536) was significantly lower upon infection with *H. pylori* wt and Δ*cagA* mutant, respectively, compared to AGS wt infected cells (Fig. 3C–E, lanes 2 and 5). These data support the hypothesis that cortactin and CagA contribute to NF-κB activation.

In line with the above results, SEAP reporter and ELISA assays revealed increased NF-κB levels and IL-8 secretion, respectively, in AGS wt cells that were infected with *H. pylori* wt for 6 h (Fig. 3F and G). Interestingly, infection with the Δ*cagA* mutant diminished NF-κB and IL-8 levels in AGS wt cells by about two-fold at this time point. Similarly, reduced levels of NF-κB activity and IL-8 secretion were observed in AGSΔ*cttn* cells infected with *H. pylori* wt and Δ*cagA* mutant, respectively (Fig. 3F and G), suggesting that CagA supports an efficient NF-κB activation and IL-8 release, which is dependent on the expression of cortactin.

### Impact of ADPH and CagA on phosphorylation of FAK, Src, IKKβ, and p65 in AGS wt vs. *cttn* knockout cells

**Figure 4.**
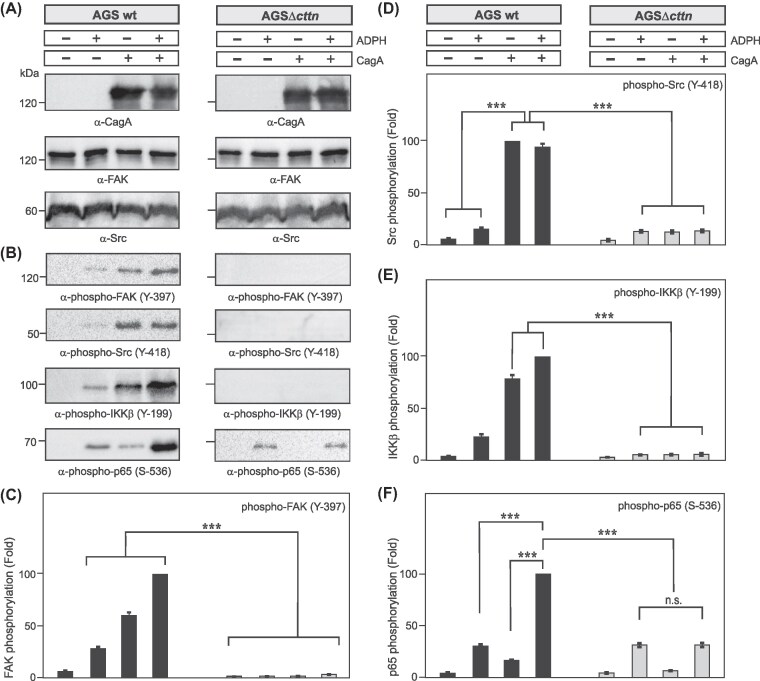
Effects of ADPH and CagA on phosphorylation of FAK, Src, IKKβ, and p65 NF-κB in AGS wt vs. cortactin knockout cells. The indicated cells were transfected with CagA and/or treated with synthetic ADPH as described in the section “Materials and methods.” (A) Protein lysates were subjected to Western blotting and probed with the indicated antibodies against CagA, total FAK, and total Src as loading controls. (B) Protein lysates from panel A were probed with the indicated phospho-specific antibodies. The blots show that phospho-FAK (Y-397) and phospho-Src (Y-418) bands are CagA-dependent and absent in Δ*cttn* cells. Activation of phospho-IKKβ (Y-199) and phospho-p65 (S-536) is strong upon CagA expression and further enhanced by combined CagA transfection and ADPH treatment. (C–F) Quantification of phospho-FAK, phospho-Src, phospho-IKKβ, and phospho-p65 band intensities shown in panel B. Mean values ± standard error are shown with significant difference *** corresponding to *P* ≤  .001.

Based on the above experiments, we proposed that ADPH is the major driver of *H. pylori*-induced NF-κB activation, while CagA may contribute by enhancing IKKβ activation via Src. We have previously shown that CagA induces cortactin activation, leading to interaction of cortactin with FAK stimulating its tyrosine phosphorylation (Tegtmeyer et al. 2011), which may control Src kinase activity and pronounced NF-κB activation. To study this possibility, AGS wt or AGSΔ*cttn* cells were transfected with CagA for 24 h and/or treated with synthetic ADPH for 45 min as shown (Fig. 4). Protein lysates were subjected to Western blotting and probed with the indicated antibodies. Blots against CagA and total FAK and Src were used as loading controls (Fig. 4A). Probing of cell lysates with phospho-specific antibodies showed that phosphorylation of FAK (Y-397), Src (Y-418), and IKKβ (Y-199) was predominantly induced in AGS wt cells and when CagA was expressed, which was strongly diminished in cortactin knockout cells treated under the same conditions (Fig. 4B–E). Remarkably, phosphorylation of p65 (S-536) in treated AGS wt cells was induced (Fig. 4B, bottom), and quantification showed that the band intensities increased significantly when both effectors (CagA and ADPH) were present (Fig. 4F). In contrast, neither purified ADPH nor ectopically expressed CagA induced detectable tyrosine phosphorylation of FAK (Y-397), Src (Y-418), or IKKβ (Y-199) in AGSΔ*cttn* cells, strongly indicating on a key role of cortactin in this process (Fig. 4B–E). Furthermore, there was only activation of p65 (S-536) in AGSΔ*cttn* cells exposed to ADPH alone or in combination with CagA, while CagA alone did not (Fig. 4B bottom and Fig. 4F). Together, these data suggest that treatment of wt cells with ADPH has a major impact on the induction of p65 phosphorylation, while CagA expression contributes to phosphorylation of p65 in an additive fashion through consecutive activation of FAK, Src, and IKKβ, all of which is dependent on the expression of cortactin.

### Effects of APDH and CagA on NF-κB activation and IL-8 production can be downregulated by knockout of *cttn, fak*, or *tifa*

**Figure 5.**
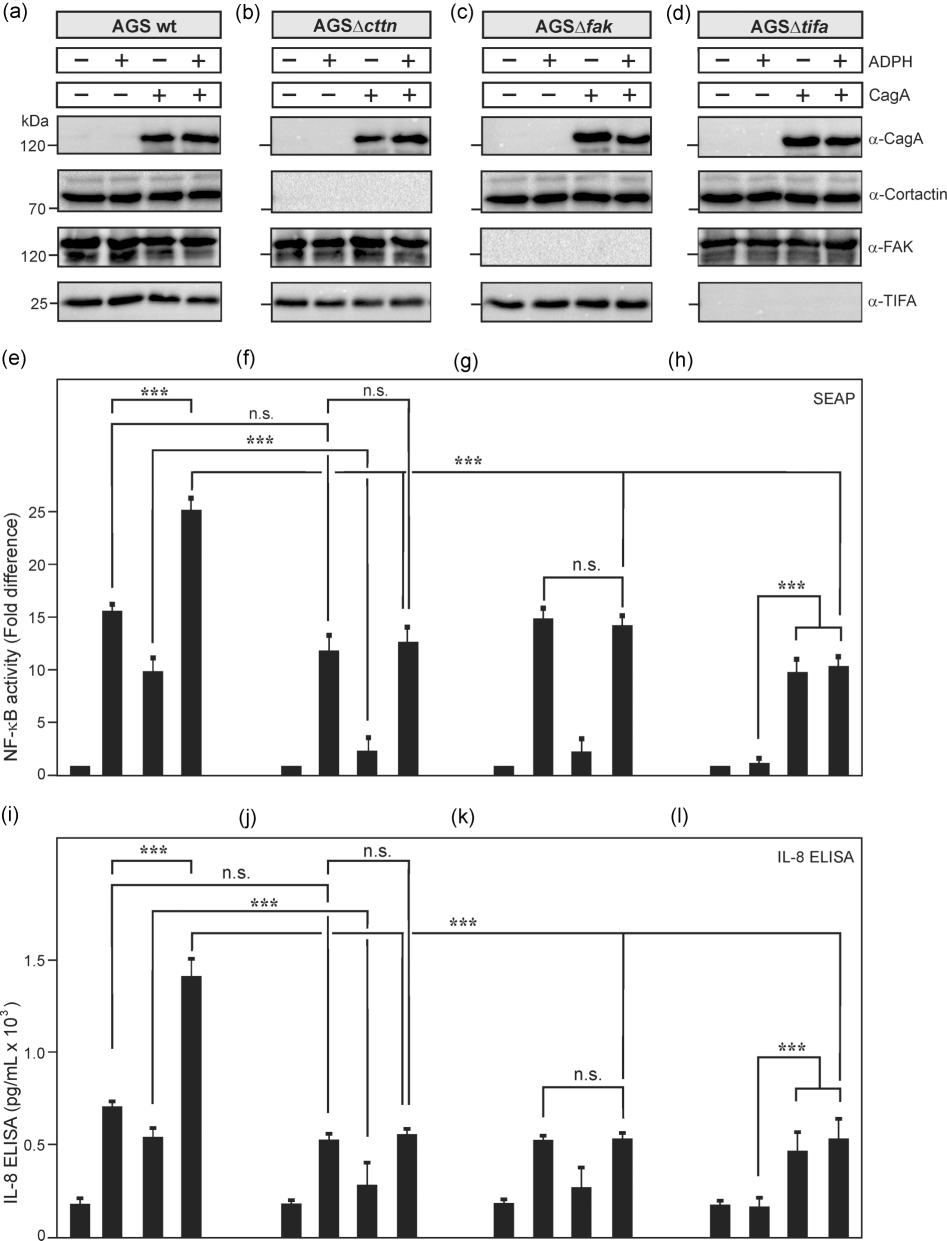
NF-κB activation in AGS wt and knockout cells for cortactin, FAK and TIFA by transfection of CagA and addition of ADPH. (A) AGS wt cells and knockout cells of (B) cortactin, (C) FAK, or (D) TIFA were transfected with CagA and/or incubated with ADPH as described in the section “Materials and methods”. Western blotting against CagA, cortactin, FAK, and TIFA confirmed similar protein expression in each sample or their corresponding absence. (E–H) Quantification of NF-κB activation by Quanti-Blue assay revealed cummulative effects of CagA and ADPH in wt cells (lane 4). Treatment of cortactin or FAK knockout cells resulted in a similar NF-κB activation by ADPH, but not CagA, while in TIFA knockout cells ADPH was unable to induce NF-κB activation showing only CagA-dependent effects. (I–L) These data correlated with the production of IL-8 as determined by standard ELISA in the same experiments. Mean values ± standard error are shown with significant difference *** corresponding to *P* ≤  .001; n.s. not significant.

In addition, we aimed to compare the above results with treated cells deficient in FAK or TIFA expression. For this purpose, AGS wt and cells with knockout in cortactin, FAK, or TIFA were transfected with CagA and/or incubated with ADPH as described above (Fig. 5). Western blotting against CagA, cortactin, FAK, and TIFA confirmed similar protein expression in each sample or their corresponding absence, respectively (Fig. 5A–D). NF-κB activation was quantified and showed enhanced effects of transfected CagA treated with ADPH in wt cells (Fig. 5E). Treatment of cortactin knockout cells resulted in a similar NF-κB activation by ADPH, but not CagA (Fig. 5F). This result was phenocopied by treated FAK knockout cells (Fig. 5G). In contrast, in TIFA knockout cells, exposure to ADPH was unable to induce NF-κB activation as expected, and thus, only effects of CagA expression are visible (Fig. 5H, lanes 3 and 4). The activation of NF-κB correlated with the amounts of IL-8 released into the cell supernatants (Fig. 5I–L). These results show that *H. pylori* ADPH and CagA can activate NF-κB and IL-8 production independent of each other, but together they are cumulative.

### NF-κB activation and KC production in MEFs are induced by expression of CagA in a cortactin-, ERK1/2 kinase-, and Src-dependent manner

**Figure 6.**
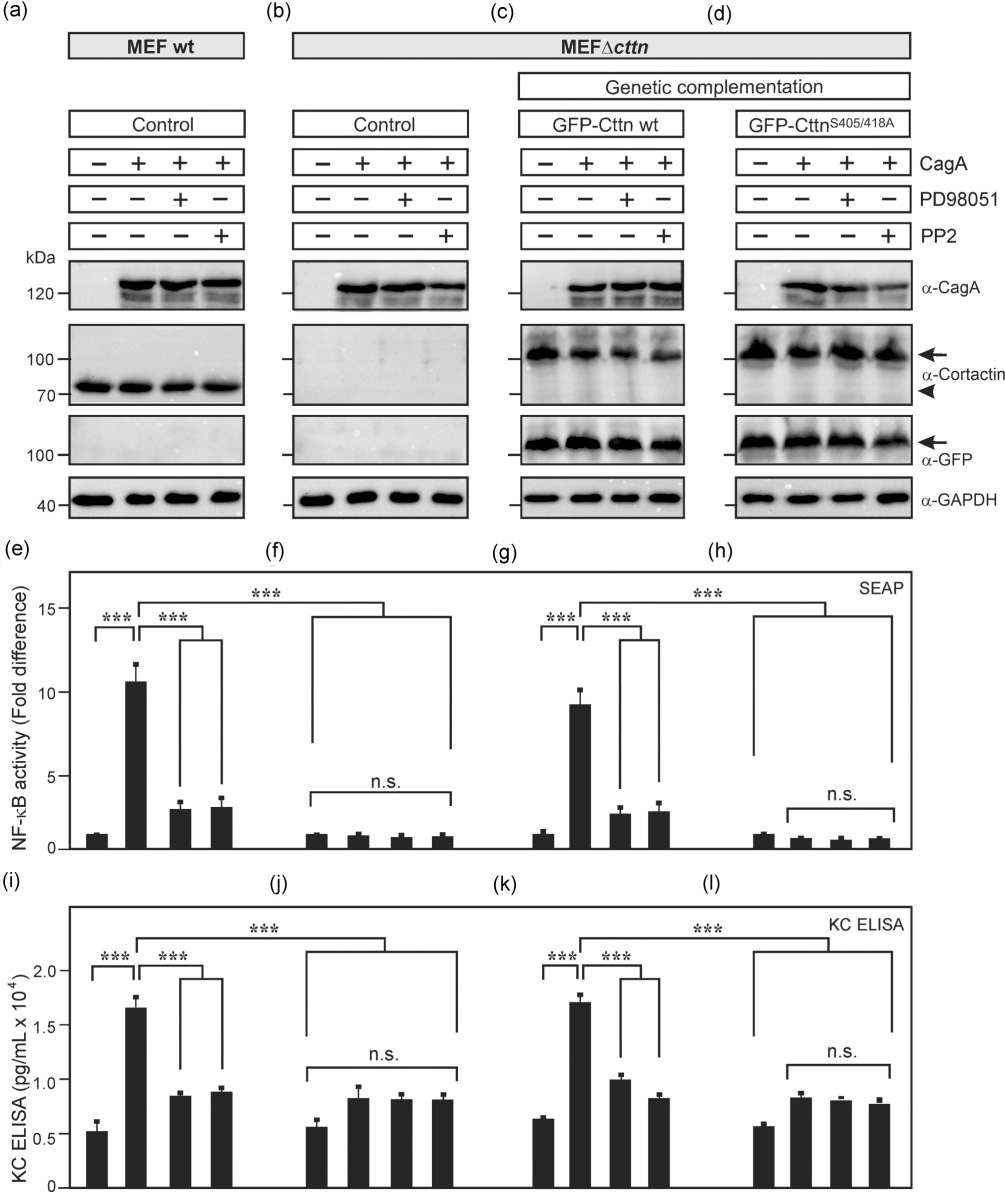
Expression of CagA promotes NF-κB activation in MEFs in a cortactin-, ERK1/2 kinase-, and Src-dependent manner. Primary MEF cells from (A) wt or (B–D) cortactin knockout mice were transfected with CagA and treated with PD98051 or PP2, pharmacological inhibitors of ERK1/2 and Src kinases, respectively. (C and D) Cortactin-deficient MEFs were additionally transfected with GFP-cortactin expression plasmids (wt or phosphorylation-deficient mutant S405/418A). (A–D) Western blotting against CagA, cortactin, GFP, and GAPDH showed similar expression of CagA and cortactin proteins in the samples. The arrow marks the GFP-cortactin fusion proteins, and the arrowhead marks endogenous cortactin proteins. (E–H) Quantification of NF-κB activity by Quanti-Blue assay after 12 h. (I–L) Quantification of KC secretion by ELISA of the respective samples shows correlation with NF-κB activation. Mean values ± standard error are shown with significant difference *** corresponding to *P* ≤  .001; n.s. not significant.

Finally, we aimed to confirm major results of the study in primary cells. For this purpose, we utilized MEF cells isolated from wt and cortactin knockout animals as described (Lai et al. 2009). These cells were transfected with CagA. Western blotting against CagA, cortactin, and GAPDH confirmed similar protein expression in each sample or corresponding absence of cortactin, respectively (Fig. 6A and B). NF-κB activation and KC (functional homolog of human IL-8 in mice; Hol et al. 2010) production were quantified and revealed induction by transfected CagA in wt cells, while pharmacological inhibition of ERK1/2 and Src kinases significantly downregulated these effects (Fig. 6E and I). In contrast, NF-κB activation and KC production by CagA were not seen in cortactin knockout MEFs (Fig. 6 F and J). These and the above experiments led us to propose that activation of Src kinase by serine phosphorylation of cortactin through ERK1/2 kinases by CagA are responsible for the NF-κB activation and KC production. To confirm this hypothesis, we cotransfected the cortactin knockout MEFs with CagA and mouse GFP-cortactin constructs (including wt and serine phosphorylation-deficient S405/418A point mutant). The results show that cotransfection of CagA with wt GFP-cortactin (Fig. 6C) restored NF-κB activation and KC production in a ERK1/2- and Src-dependent fashion (Fig. 6G and K), whereas expression of the GFP-cortactin S405/418A mutant did not (Fig. 6H and L). These data demonstrate that *H. pylori* CagA can activate NF-κB and KC release, which depends on cortactin serine phosphorylation by ERK1/2 and Src kinase activity.

## Discussion

**Figure 7.**
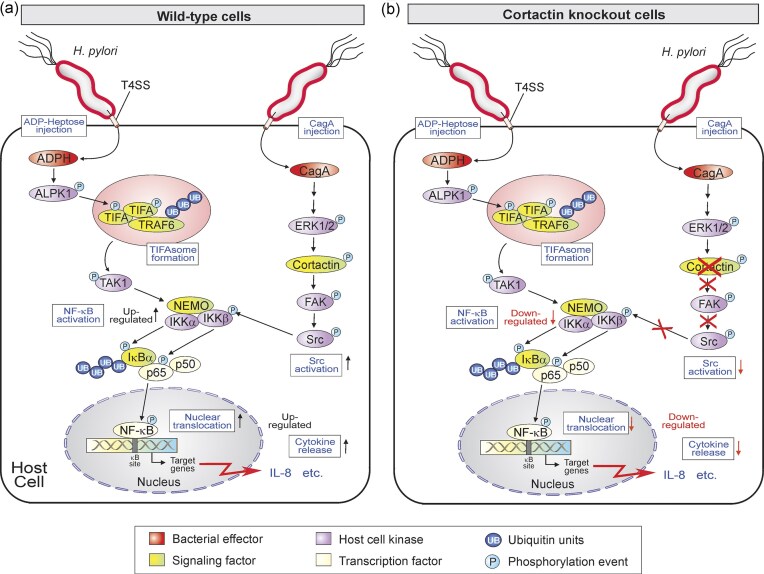
Model of NF-κB signaling induced by *H. pylori* in (A) wt or (B) Δ*cttn* knockout cells. The *H. pylori* T4SS can inject two major substrates, CagA and ADPH, with the latter being responsible for the main portion of proinflammatory signaling through transcription factor NF-κB. In this scenario, CagA appears as a “helping molecule” by enhancing the ADPH response. In particular, translocated ADPH binds to a novel intracellular pathogen recognition receptor (PRR), cytoplasmic alpha kinase-1 (ALPK1). ALPK1 becomes autophosphorylated and then phosphorylates the adapter protein TIFA. TIFA then oligomerizes and forms the so-called TIFAsome. The activated TIFAsome in turn activates TAK kinase and the IKK complex. This IKK complex controls the downstream NF-κB complex, and p65 further translocates into the nucleus and activates IL-8 gene transcription. We hypothesize that injected CagA exhibits a supporting function in this signaling by stimulating ERK1/2-mediated serine phosphorylation of cortactin and tyrosine phosphorylation of FAK (Tegtmeyer et al. 2011). FAK and Src can interact and further activate each other. Active Src can then phosphorylate IKKβ (Y-199), which fully activates the IKK complex and overall NF-κB activity. When cortactin is absent, as in AGS or MEF Δ*cttn* knockout cells, NF-κB activity is downregulated by the portion contributed by CagA>cortactin>FAK>Src>IKKβ-mediated signaling as indicated in panel B.

*Helicobacter pylori* infection is well-known to induce proinflammatory IL-8 secretion in a NF-κB-dependent manner (Brandt et al. 2005, Sokolova et al. 2013). Two T4SS-dependent bacterial factors, ADPH and CagA, are involved in the activation of these signaling cascades (Naumann et al. 2023). In particular, ADPH targets the ALPK>TIFA>TRAF6 cascade followed by activation of the canonical NF-κB pathway (Gall et al. 2017, Stein et al. 2017, Zimmermann et al. 2017, Zhou et al. 2018, Pfannkuch et al. 2019, Maubach et al. 2021). On the other hand, CagA-dependent stimulation of NF-κB signaling was described to involve the CagA>Ras>Raf>MEK>ERK1/2 pathway with a yet unknown link from ERK1/2 to the NF-κB cascade (Brandt et al. 2005). In the present work, we were able to decipher how the two ADPH- and CagA-dependent pathways costimulate NF-κB signaling upon *H. pylori* infection and ADPH/CagA treatment with cortactin as a connecting link (summarized in a model, Fig. 7).

A key cytoskeletal regulatory protein, cortactin, is well-known to regulate multiple cellular functions including cell migration, GTPase signaling, vesicular trafficking, as well as being implicated in various diseases such as lung disorders and cancer (Schnoor et al. 2018, Tegtmeyer et al. 2021, Bandela et al. 2022). In the context of host–pathogen interactions, cortactin appears as a major target of *H. pylori*, which enables the bacterium to modulate host actin-cytoskeletal rearrangements as well as cell motility and adhesion (Sharafutdinov et al. 2020a, b, Selbach and Backert 2005, Tegtmeyer et al. 2011). Surprisingly, we recently identified a completely new function of cortactin in the *H. pylori*-, *S. enterica-*, and *P. aeruginosa-*induced NF-κB signaling (Tegtmeyer et al. 2022). Here, we further expand our understanding on the *H. pylori*-induced NF-κB signal transduction cascade, by providing a link to Src kinase, which is required for the efficient phosphorylation of Y-199 in IKKβ, a key molecule of canonical NF-κB activation. We explicitly show by immunofluorescence microscopy that cortactin is necessary for effective nuclear translocation of p65 NF-κB and IL-8 production upon infection by *H. pylori* (Figs 1 and 2). Deletion of the cortactin gene in AGS cells by CRISPR-Cas9 diminished *H. pylori*-induced nuclear translocation of phospho-p65 and IL-8 expression, but did not completely abrogated this response (Figs 1 and 2). Therefore, we propose that the portion of active p65 and IL-8 expression in cortactin-deficient cells can be explained by the activity of another potent NF-κB activator, ADPH. ADPH was shown to activate NF-κB signaling via activation of the ALPK1>TIFA pathway as discussed above (Zhou et al. 2018).

We next wanted to identify whether the portion of cortactin-dependent NF-κB activation requires ADPH or CagA. Interestingly, our experiments showed that cortactin is not involved in the ADPH-dependent TIFAsome activation, since phosphorylation of TIFA (T-9) was similarly strong both in infected AGS wt and cortactin-deficient cells (Fig. 3A and B). However, the downstream IKKβ and p65 phosphorylation was diminished in infected cortactin deficient cells. Furthermore, similarly reduced IKKβ and p65 phosphorylation levels were observed in AGS wt cells infected with Δ*cagA* mutant *H. pylori*. These data strongly suggest that IKKβ phosphorylation appears independently via two signaling pathways, initiated by ADPH>TIFA and CagA>cortactin, which together result in elevated NF-κB activity and IL-8 release (Figs 3–5).

We previously showed that cortactin is required for efficient CagA tyrosine phosphorylation via Src activation, while expression of constitutively active Src promoted NF-κB activity and IL-8 secretion in a cortactin-dependent manner (Knorr et al. 2021, Tegtmeyer et al. 2022). Mechanistically, it was previously shown that *H. pylori* infection can transiently induce Src binding to IKKβ, which resulted in efficient phosphorylation of IκBα, followed by the release of p65 from the inhibitory complex (Rieke et al. 2011). We therefore proposed that cortactin is required for efficient Src activation, which leads to IKKβ activation upon *H. pylori* infection. Importantly, neither ADPH nor CagA were able to trigger the phosphorylation of FAK (Y-397) and Src (Y-418) in cortactin-deficient cells. Therefore, CagA/cortactin-induced activation of Src is required for phosphorylation of IKKβ (Y-199) and subsequently p65 (S-536) (Fig. 7A and B). Thus, our findings are in well agreement with the above mentioned study by Rieke et al. (2011). In addition, our results on reduced NF-κB activation in cortactin knockout cells was phenocopied in treated FAK knockout cells (Fig. 5). This finding matches well with the long known interaction of FAK with Src and their mutual activation mechanism (Schaller et al. 1994, Calalb et al. 1995). Since cortactin can also interact with and stimulate FAK (Tegtmeyer et al. 2011), we propose that a cortactin>FAK>Src signaling complex could accelerate IKKβ phosphorylation and subsequent NF-κB activation in the context of *H. pylori* infection. In agreement with this hypothesis, we found that expression of CagA in MEFs induced NF-κB activation and cytokine release in a manner dependent on cortactin serine phosphorylation by ERK1/2 and downstream Src activation (Fig. 6).

Taken together, we suggest that in *H. pylori* infection, ADPH is responsible for a major portion of proinflammatory NF-κB activation, while coactivation by CagA is required for full NF-κB activity (Fig. 7A and B). In line with this assumption, our infection experiments with *H. pylori* wt compared to Δ*gmhA* and Δ*cagA* mutants showed a higher influence of ADPH on NF-κB activation (Fig. 3). Furthermore, assessment of NF-κB activity induced by purified ADPH, ectopically expressed CagA or their combination, revealed that ADPH is a stronger inducer than CagA, while the highest activation was observed when both ADPH and CagA were present (Fig. 5A and E). ADPH is an emerging bacterial factor, identified in recent years as a new PAMP of various pathogens (Zhou et al. 2018, Pfannkuch et al. 2019, García-Weber and Arrieumerlou 2021). In the current study, we showed how two *H. pylori* T4SS factors, ADPH and CagA, can modulate two independent pathways, merging together to promote proinflammatory NF-κB signaling. We identified cortactin as a major host factor mediating CagA-dependent FAK>Src>IKKβ pathway activation. Future studies should address whether the identified pathways might be targeted for improved therapies of *H. pylori*-related gastric diseases.

## Supplementary Material

uqaf049_Supplemental_File

## References

[bib1] Ando T, Perez-Perez GI, Kusugami K et al. Anti-CagA immunoglobulin G responses correlate with interleukin-8 induction in human gastric mucosal biopsy culture. Clin Diagn Lab Immunol. 2000;7:803–9. 10.1128/CDLI.7.5.803-809.2000. 10973458 PMC95959

[bib2] Backert S, Tegtmeyer N. Type IV secretion and signal transduction of *Helicobacter pylori* CagA through interactions with host cell receptors. Toxins. 2017;9:115. 10.3390/toxins9040115. 28338646 PMC5408189

[bib3] Bandela M, Belvitch P, Garcia J et al. Cortactin in lung cell function and disease. Int J Mol Sci. 2022;23:4606. 10.3390/ijms23094606. 35562995 PMC9101201

[bib4] Blumenthal B, Hoffmann C, Aktories K et al. The cytotoxic necrotizing factors from *Yersinia pseudotuberculosis* and from *Escherichia coli* bind to different cellular receptors but take the same route to the cytosol. Infect Immun. 2007;75:3344–53. 10.1128/IAI.01937-06. 17438028 PMC1932955

[bib5] Brandt S, Kwok T, Hartig R et al. NF-kappa B activation and potentiation of proinflammatory responses by the *Helicobacter pylori* CagA protein. Proc Natl Acad Sci USA. 2005;102:9300–5. 10.1073/pnas.0409873102. 15972330 PMC1166591

[bib6] Calalb MB, Polte TR, Hanks SK. Tyrosine phosphorylation of focal adhesion kinase at sites in the catalytic domain regulates kinase activity: a role for Src family kinases. Mol Cell Biol. 1995;15:954–63. 10.1128/MCB.15.2.954. 7529876 PMC231984

[bib7] Chauhan N, Tay ACY, Marshall BJ et al. *Helicobacter pylori* VacA, a distinct toxin exerts diverse functionalities in numerous cells: an overview. Helicobacter. 2019;24:e12544. 10.1111/hel.12544. 30324717

[bib8] Chen L, Greene W. Shaping the nuclear action of NF-κB. Nat Rev Mol Cell Biol. 2004;5:392–401. 10.1038/nrm1368. 15122352

[bib9] Crabtree JE, Xiang Z, Lindley IJ et al. Induction of interleukin-8 secretion from gastric epithelial cells by a *cagA* negative isogenic mutant of *Helicobacter pylori*. J Clin Pathol. 1995;48:967–9. 10.1136/jcp.48.10.967. 8537502 PMC502959

[bib10] Davuluri G, Augoff K, Schiemann W et al. WAVE3-NFκB interplay is essential for the survival and invasion of cancer cells. PLoS One. 2014;9:e110627. 10.1371/journal.pone.0110627. 25329315 PMC4199728

[bib11] Dragneva Y, Anuradha CD, Valeva A et al. Subcytocidal attack by staphylococcal alpha-toxin activates NF-kappaB and induces interleukin-8 production. Infect Immun. 2001;69:2630–5. 10.1128/IAI.69.4.2630-2635.2001. 11254628 PMC98200

[bib12] Gall A, Gaudet RG, Gray-Owen SD et al. TIFA signaling in gastric epithelial cells initiates the *cag* type 4 secretion system-dependent innate immune response to *Helicobacter pylori* infection. mBio. 2017;8:e01168–17. 10.1128/mBio.01168-17. 28811347 PMC5559637

[bib13] García-Weber D, Arrieumerlou C. ADP-heptose: a bacterial PAMP detected by the host sensor ALPK1. Cell Mol Life Sci. 2021;78:17–29. 10.1007/s00018-020-03577-w. 32591860 PMC11072087

[bib14] Gong M, Ling S, Lui S et al. *Helicobacter pylori* γ-glutamyl transpeptidase is a pathogenic factor in the development of peptic ulcer disease. Gastroenterology. 2010;139:564–73. 10.1053/j.gastro.2010.03.050. 20347814

[bib15] Graham D, Miftahussurur M. *Helicobacter pylori* urease for diagnosis of *Helicobacter pylori* infection: a mini review. J Adv Res. 2018;13:51–57. 10.1016/j.jare.2018.01.006. 30094082 PMC6077137

[bib16] Hatakeyama M. Malignant *H. pylori*-associated diseases: gastric cancer and MALT lymphoma. Adv Exp Med Biol. 2019;1149:135–49. 10.1007/5584_2019_363. 31016622

[bib17] Hol J, Wilhelmsen L, Haraldsen G. The murine IL-8 homologues KC, MIP-2, and LIX are found in endothelial cytoplasmic granules but not in Weibel-Palade bodies. J Leukocyte Biol. 2010;87:501–8. 10.1189/jlb.0809532. 20007247

[bib18] Huang W, Chen J, Chen C. c-Src-dependent tyrosine phosphorylation of IKKβ is involved in tumor necrosis factor-α-induced intercellular adhesion molecule-1 expression. J Biol Chem. 2003;278:9944–52. 10.1074/jbc.M208521200. 12645577

[bib19] Keates S, Hitti Y, Upton M et al. *Helicobacter pylori* infection activates NF-kappa B in gastric epithelial cells. Gastroenterology. 1997;113:1099–109. 10.1053/gast.1997.v113.pm9322504. 9322504

[bib20] Keates S, Keates AC, Warny M et al. Differential activation of mitogen-activated protein kinases in AGS gastric epithelial cells by cag+ and *cag*- *Helicobacter pylori*. J Immunol. 1999;163: 5552–9. 10.4049/jimmunol.163.10.5552. 10553083

[bib21] Kim SY, Lee YC, Kim HK et al. *Helicobacter pylori* CagA transfection of gastric epithelial cells induces interleukin-8. Cell Microbiol. 2006;8:97–106. 10.1111/j.1462-5822.2005.00603.x. 16367869

[bib22] Knorr J, Sharafutdinov I, Fiedler F et al. Cortactin is required for efficient Fak, Src and abl tyrosine kinase activation and phosphorylation of *Helicobacter pylori* CagA. Int J Mol Sci. 2021;22:6045. 10.3390/ijms22116045. 34205064 PMC8199859

[bib23] Lai FP, Szczodrak M, Oelkers JM et al. Cortactin promotes migration and platelet-derived growth factor-induced actin reorganization by signaling to Rho-GTPases. MBoC. 2009;20:3209–23. 10.1091/mbc.e08-12-1180. 19458196 PMC2710823

[bib24] Lim M, Maubach G, Naumann M. CYLD-TRAF6 interaction promotes ADP-heptose-induced NF-κb signaling in *H. pylori* infection. EMBO Rep. 2025;26:3241–63., 10.1038/s44319-025-00480-y. 40404856 PMC12238516

[bib25] Loh JT, Torres VJ, Algood HMS et al. *Helicobacter pylori* HopQ outer membrane protein attenuates bacterial adherence to gastric epithelial cells. FEMS Microbiol Lett. 2008;289:53–58. 10.1111/j.1574-6968.2008.01368.x. 19065710 PMC2651568

[bib26] Maubach G, Lim M, Sokolova O et al. TIFA has dual functions in *Helicobacter pylori*-induced classical and alternative NF-κb pathways. EMBO Rep. 2021;22:e52878. 10.15252/embr.202152878. 34328245 PMC8419686

[bib27] Milivojevic M, Dangeard A, Kasper C et al. ALPK1 controls TIFA/TRAF6-dependent innate immunity against heptose-1,7-bisphosphate of gram-negative bacteria. PLoS Pathog. 2017;13:e1006224. 10.1371/journal.ppat.1006224. 28222186 PMC5336308

[bib28] Moese S, Selbach M, Zimny-Arndt U et al. Identification of a tyrosine-phosphorylated 35 kDa carboxy-terminal fragment (p35CagA) of the *Helicobacter pylori* CagA protein in phagocytic cells: processing or breakage?. Proteomics. 2001;1:618–29. 10.1002/1615-9861(200104)1:4<618::AID-PROT618>3.0.CO;2-C. 11681214

[bib29] Mueller D, Tegtmeyer N, Brandt S et al. c-Src and c-abl kinases control hierarchic phosphorylation and function of the CagA effector protein in Western and East Asian *Helicobacter pylori* strains. J Clin Invest. 2012;122:1553–66. 10.1172/JCI61143. 22378042 PMC3314471

[bib30] Naumann M, Ferino L, Sharafutdinov I et al. Gastric epithelial barrier disruption, inflammation and oncogenic signal transduction by *Helicobacter pylori*. Curr Top Microbiol Immunol. 2023;444:207–38. 10.1007/978-3-031-47331-9_8. 38231220

[bib31] Nozawa Y, Nishihara K, Peek RM et al. Identification of a signaling cascade for interleukin-8 production by *Helicobacter pylori* in human gastric epithelial cells. Biochem Pharmacol. 2002;64:21–30. 10.1016/s0006-2952(02)01030-4. 12106602

[bib32] Pachathundikandi SK, Tegtmeyer N, Arnold IC et al. T4SS-dependent TLR5 activation by *Helicobacter pylori* infection. Nat Commun. 2019;10:5717. 10.1038/s41467-019-13506-6. 31844047 PMC6915727

[bib33] Papadakos KS, Sougleri IS, Mentis AF et al. Presence of terminal EPIYA phosphorylation motifs in *Helicobacter pylori* CagA contributes to IL-8 secretion, irrespective of the number of repeats. PLoS One. 2013;8:e56291. 10.1371/journal.pone.0056291. 23409168 PMC3567036

[bib34] Pfannkuch L, Hurwitz R, Traulsen J et al. ADP heptose, a novel pathogen-associated molecular pattern identified in *Helicobacter pylori*. FASEB J. 2019;33:9087–99. 10.1096/fj.201802555R. 31075211 PMC6662969

[bib35] Rieke C, Papendieck A, Sokolova O et al. *Helicobacter pylori*-induced tyrosine phosphorylation of ikkβ contributes to NF-κb activation. Biol Chem. 2011;392:387–93. 10.1515/BC.2011.029. 21294676

[bib36] Schaller MD, Hildebrand JD, Shannon JD et al. Autophosphorylation of the focal adhesion kinase, pp125FAK, directs SH2-dependent binding of pp60src. Mol Cell Biol. 1994;14:1680–8. 10.1128/mcb.14.3.1680-1688.1994. 7509446 PMC358526

[bib37] Schindelin J, Arganda-Carreras I, Frise E et al. Fiji: an open-source platform for biological-image analysis. Nat Methods. 2012;9:676–82. 10.1038/nmeth.2019. 22743772 PMC3855844

[bib38] Schnoor M, Stradal TE, Rottner K. Cortactin: cell functions of A multifaceted actin-binding protein. Trends Cell Biol. 2018;28:79–98. 10.1016/j.tcb.2017.10.009. 29162307

[bib39] Selbach M, Backert S. Cortactin: an Achilles' heel of the actin cytoskeleton targeted by pathogens. Trends Microbiol. 2005;13:181–9. 10.1016/j.tim.2005.02.007. 15817388

[bib40] Sharafutdinov I, Backert S, Tegtmeyer N. Cortactin: a major cellular target of the Gastric carcinogen *Helicobacter pylori*. Cancers. 2020b;12:159. 10.3390/cancers12010159. 31936446 PMC7017262

[bib41] Sharafutdinov I, Esmaeili DS, Harrer A et al. *Campylobacter jejuni* serine protease HtrA cleaves the tight junction component claudin-8. Front Cell Infect Microbiol. 2020a;10:590186. 10.3389/fcimb.2020.590186. 33364202 PMC7752809

[bib42] Sharafutdinov I, Friedrich B, Rottner K et al. Cortactin: a major cellular target of viral, protozoal, and fungal pathogens. Mol Microbiol. 2024;122:165–83. 10.1111/mmi.15284. 38868928

[bib43] Sharafutdinov I, Harrer A, Müsken M et al. Cortactin-dependent control of Par1b-regulated epithelial cell polarity in *Helicobacter* infection. Cell Insight. 2024;3:100161. 10.1016/j.cellin.2024.100161. 38646547 PMC11033139

[bib44] Sharafutdinov I, Knorr J, Rottner K et al. Cortactin: a universal host cytoskeletal target of gram-negative and gram-positive bacterial pathogens. Mol Microbiol. 2022;118:623–36. 10.1111/mmi.15002. 36396951

[bib45] Sharma SA, Tummuru MK, Miller GG et al. Interleukin-8 response of gastric epithelial cell lines to *Helicobacter pylori* stimulation in vitro. Infect Immun. 1995;63:1681–7. 10.1128/iai.63.5.1681-1687.1995. 7729872 PMC173210

[bib46] Snelling T, Shpiro N, Gourlay R et al. Co-ordinated control of the ADP-heptose/ALPK1 signalling network by the E3 ligases TRAF6, TRAF2/c-IAP1 and LUBAC. Biochem J. 2022;479:2195–216. 10.1042/BCJ20220401. 36098982 PMC9704527

[bib47] Sokolova O, Borgmann M, Rieke C et al. *Helicobacter pylori* induces type 4 secretion system-dependent, but CagA-independent activation of iκbs and NF-κb/RelA at early time points. Int J Med Microbiol. 2013;303:548–52. 10.1016/j.ijmm.2013.07.008. 23972614

[bib48] Sokolova O, Naumann M. NF-κb signaling in gastric cancer. Toxins. 2017;9:119. 10.3390/toxins9040119. 28350359 PMC5408193

[bib49] Stein SC, Faber E, Bats SH et al. *Helicobacter pylori* modulates host cell responses by CagT4SS-dependent translocation of an intermediate metabolite of LPS inner core heptose biosynthesis. PLoS Pathog. 2017;13:e1006514. 10.1371/journal.ppat.1006514. 28715499 PMC5531669

[bib50] Stephenson A, Taggart D, Xu G et al. The inhibitor of κb kinase β (IKKβ) phosphorylates iκbα twice in a single binding event through a sequential mechanism. J Biol Chem. 2023;299:102796. 10.1016/j.jbc.2022.102796. 36528060 PMC9843440

[bib51] Stingl K, Altendorf K, Bakker E. Acid survival of *Helicobacter pylori*: how does urease activity trigger cytoplasmic pH homeostasis?. Trends Microbiol. 2002;10:70–74. 10.1016/S0966-842X(01)02287-9. 11827807

[bib52] Su YL, Huang HL, Huang BS et al. Combination of OipA, BabA, and SabA as candidate biomarkers for predicting *Helicobacter pylori*-related gastric cancer. Sci Rep. 2016;6:12. 10.1038/srep36442. 27819260 PMC5098209

[bib53] Tegtmeyer N, Harrer A, Rottner K et al. *Helicobacter pylori* CagA induces cortactin Y-470 phosphorylation-dependent gastric epithelial cell scattering via abl, Vav2 and Rac1 activation. Cancers. 2021;13:4241. 10.3390/cancers13164241. 34439396 PMC8391897

[bib54] Tegtmeyer N, Neddermann M, Lind J et al. Toll-like receptor 5 activation by the CagY repeat domains of *Helicobacter pylori*. Cell Rep. 2020;32:1345–58. 10.1016/j.celrep.2020.108159. 32937132

[bib55] Tegtmeyer N, Rivas Traverso F, Rohde M et al. Electron microscopic, genetic and protein expression analyses of *Helicobacter acinonychis* strains from a Bengal tiger. PLoS One. 2013;8:e71220. 10.1371/journal.pone.0071220. 23940723 PMC3733902

[bib56] Tegtmeyer N, Soltan Esmaeili D, Sharafutdinov I et al. Importance of cortactin for efficient epithelial NF-kB activation by *Helicobacter pylori, Salmonella enterica* and *Pseudomonas aeruginosa*, but not *Campylobacter* spp. EuJMI. 2022;11:95–103. 10.1556/1886.2021.00023. PMC8830411 35060920

[bib57] Tegtmeyer N, Wittelsberger R, Hartig R et al. Serine phosphorylation of cortactin controls focal adhesion kinase activity and cell scattering induced by *Helicobacter pylori*. Cell Host Microbe. 2011;9:520–31. 10.1016/j.chom.2011.05.007. 21669400

[bib58] Yamaoka Y, Kita M, Kodama T et al. *Helicobacter pylori cagA* gene and expression of cytokine messenger RNA in gastric mucosa. Gastroenterology. 1996;110:1744–52. 10.1053/gast.1996.v110.pm8964399. 8964399

[bib59] Zhang XS, Tegtmeyer N, Traube L et al. A specific A/T polymorphism in western tyrosine phosphorylation B-motifs regulates *Helicobacter pylori* CagA epithelial cell interactions. PLoS Pathog. 2015;11:e1004621. 10.1371/journal.ppat.1004621. 25646814 PMC4412286

[bib60] Zhou P, She Y, Dong N et al. Alpha-kinase 1 is a cytosolic innate immune receptor for bacterial ADP-heptose. Nature. 2018;561:122–6. 10.1038/s41586-018-0433-3. 30111836

[bib61] Zimmermann S, Pfannkuch L, Al-Zeer M et al. ALPK1-and TIFA-dependent Innate immune response triggered by the *Helicobacter pylori* type IV secretion system. Cell Rep. 2017;20:2384–95. 10.1016/j.celrep.2017.08.039. 28877472

