# Supplementary figures and images for: Enhanced ADP-heptose-dependent NF-κB activation by Helicobacter pylori CagA through cortactin-Src-dependent tyrosine phosphorylation of IKKβ
Source: Microlife. 2026 Jan 6;7:uqaf049. doi: 10.1093/femsml/uqaf049 (PMC12850538; doi:10.1093/femsml/uqaf049)

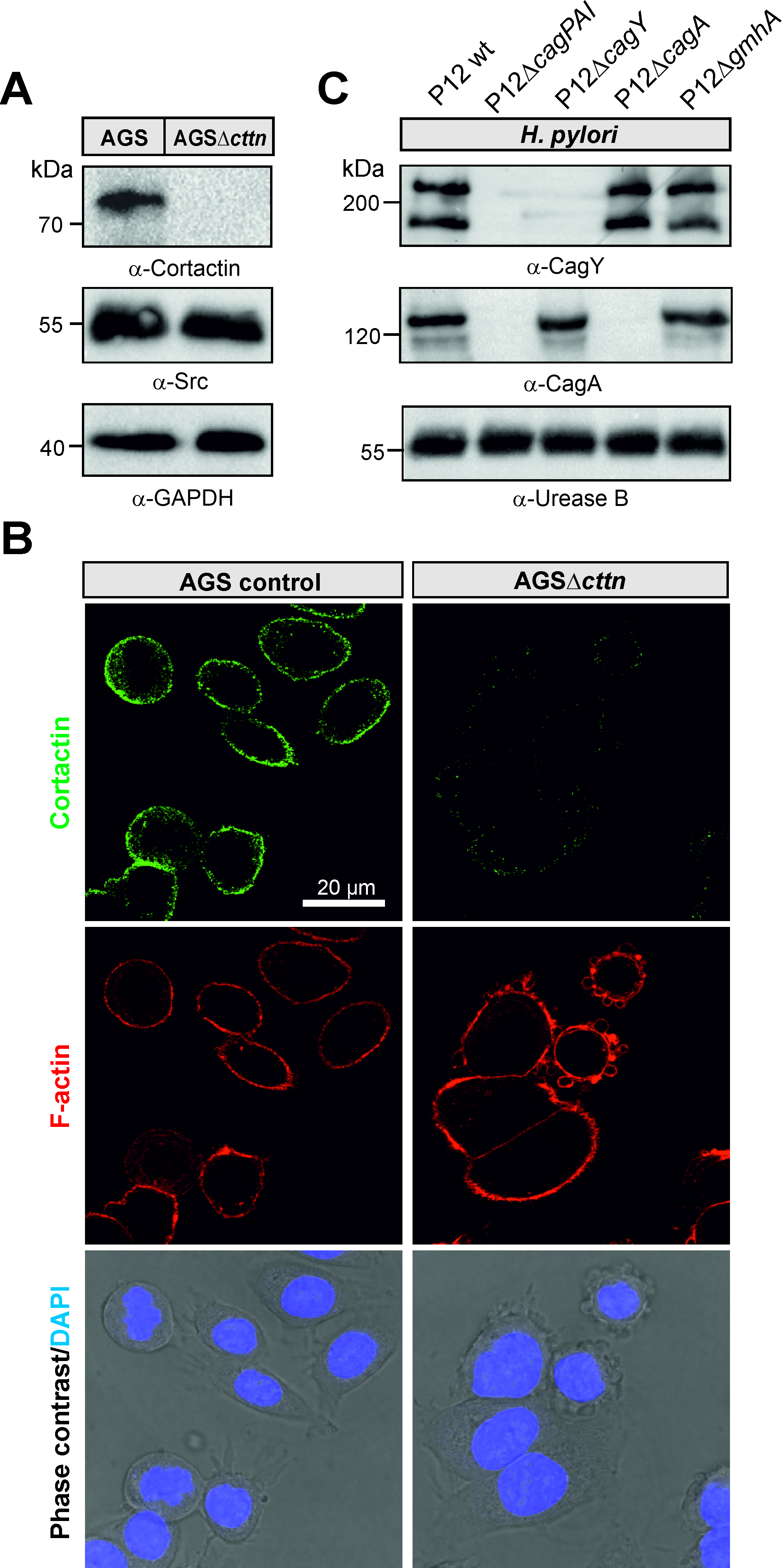

Supplement: uqaf049_Supplemental_File [file uqaf049_supplemental_file.zip › Supplemental Figure S1.tif]
